# MRI plaque imaging and its role in population-based studies

**DOI:** 10.1186/1741-7015-8-78

**Published:** 2010-11-30

**Authors:** Chun Yuan, Jarett D Berry

**Affiliations:** 1 Department of Radiology, University of Washington School of Medicine, Seattle, WA, USA; 2 Department of Internal Medicine-Cardiology, University of Texas Southwestern Medical School, Dallas, TX, USA

## Abstract

Noninvasive direct vessel wall (plaque) imaging may provide a good opportunity to study unique aspects of atherosclerotic lesions in different populations. The article published by Esposito *et al*. provides new insights into our understanding of diabetic atherosclerotic vascular disease by using direct plaque imaging techniques. The findings from this article call for attention to more *in vivo *imaging to understand the nature of high-risk atherosclerosis, especially in prospective studies in diabetic patients.

See research article: http://www.biomedcentral.com/1471-2342/10/27/abstract

## 

In an article published this month in *BMC Medical Imaging*, Esposito *et al*. [1] provide an important contribution to our understanding of diabetic atherosclerotic vascular disease by applying direct atherosclerotic plaque imaging by magnetic resonance imaging (MRI) to a large group of patients with and without diabetes. In this study, the authors observed an association between diabetes and high-risk carotid plaque characteristics. The authors also observed that carotid plaques may be more in danger of rupturing during endarterectomy than plaques of nondiabetic patients, leading to a greater propensity for distal embolization into the cerebral vasculature.

The effect of diabetes on the atherosclerotic process has been well studied in the past, with multiple studies demonstrating marked increases in the presence of subclinical atherosclerosis across all multiple vascular beds in diabetic patients [2-5]. However, most conventional imaging modalities such as coronary artery calcium and carotid intimal media thickness (IMT) provide quantitative measures of atherosclerosis with limited insight into well-established complexities of the atherosclerotic process, such as the formation of complex, high-risk plaques [6].

More recently, novel MRI-based techniques have been established which can identify high-risk carotid plaques through the detection of major tissue components such as lipid-rich necrotic core, intraplaque hemorrhage and calcium [7-9]. These techniques represent a reliable measure of these plaque features as compared to histology and have been shown to represent significant determinants of both clinical (i.e., stroke) [10] and subclinical (i.e., plaque progression) events [11]. Although these techniques have been well studied in clinical populations, there has been relatively little application of these imaging techniques to diabetic atherosclerotic disease in particular. Therefore, the findings by Esposito and colleagues [1] represent an important contribution to our understanding of the topic, suggesting that carotid atherosclerosis in diabetic patients is qualitatively distinct.

There are mechanistic observations to suggest the unique characteristics of diabetic atherosclerosis [12-14]. For example, high levels of circulating glucose have been associated with a decrease in nitric oxide availability in the endothelium, thereby translating into atherosclerotic plaque formation [15]. Additional observations have suggested that an increased formation of reactive oxygen species in diabetic patients leads to downstream changes in the formation of inflammatory proteins [16]. Thus, there is biologic plausibility to the argument that diabetic atherosclerosis might in fact be qualitatively different from nondiabetic atherosclerosis.

Some prior studies [17], but not all [18], have observed significant differences in the characteristics of atherosclerotic plaque among individuals with diabetes as compared to individuals without diabetes. For example, in a study of 270 autopsy cases, the presence of diabetes was associated with larger necrotic cores, a greater burden of inflammatory infiltrate and a higher prevalence of plaque rupture [17]. However, the presence of diabetes was also associated with marked differences in the overall quantitative burden of atherosclerosis, suggesting the importance of both quantitative and qualitative differences in diabetic atherosclerotic disease.

Prior studies in the carotid MRI literature also observed the strong correlation between the quantitative burden of atherosclerosis and high-risk plaque features such as lipid-rich necrotic core and intraplaque hemorrhage. For example, in the ARIC Carotid MRI study [19], the prevalence of lipid-rich necrotic core was relatively common among participants with a carotid wall thickness of ≥1.5 mm (42% prevalence), in contrast to those participants with a wall thickness of <1.5 mm (0.6% prevalence). Similar findings were observed in a separate study demonstrating the importance of plaque burden in the development of intraplaque hemorrhage [20].

Thus, the strong correlation between plaque burden and high-risk plaque characteristics observed in other studies is important to consider in evaluating the current study by Esposito and colleagues [1]. Although the presence of diabetes was associated with a higher prevalence of high-risk plaque characteristics, the authors provided no information on the overall plaque burden between these two groups (i.e., maximum wall thickness). Without these data, our ability to interpret these findings is limited.

Finally, the Esposito *et al*. [1] study also observed an association between diabetes and the prevalence of distal embolization in the cerebral vasculature after carotid endarterectomy, suggesting the hypothesis that the presence of diabetes might have clinical implications for the treatment of carotid disease. Several cautionary points should be noted in the interpretation of these results. First, diabetes was significantly associated with a symptomatic presentation of carotid artery disease (39% *vs*. 22.5%; *P *= 0.02). Although the authors did adjust for the presence of symptoms in their analyses, the small number of patients undergoing surgery (*N *= 72) results in a point estimate with wide confidence intervals which should be interpreted with caution. Second, the authors do not include age as a covariate in these analyses. Because we are not given the association between age and diabetes among those referred for surgery, the relative importance of age and diabetes in influencing these findings cannot be adequately assessed.

In summary, because direct plaque imaging by MRI can characterize both lesion size and composition, this technique represents a unique opportunity to broaden our understanding of atherosclerotic vascular disease in different clinical populations (i.e., diabetes). Such studies are not only safe and feasible [21] but also can be extended into prospective study designs which are critically needed to improve our understanding of atherosclerosis progression and the development of high-risk lesions.

## Competing interests

The corresponding author is the recipient of grants from the National Institutes of Health, VP Diagnostics and Philips Medical, as well as nonfinancial competing interest as a member of the advisory board of BG Medicine.

## Authors' contributions

The authors contributed equally to the writing of this commentary.

## Pre-publication history

The pre-publication history for this paper can be accessed here:

http://www.biomedcentral.com/1741-7015/8/78/prepub
